# *QuickStats*: Percentage* of Adults Aged ≥18 Years with Kidney Disease,^†^ by Age Group and Sex — National Health Interview Survey,^§^ United States, July–December 2020

**DOI:** 10.15585/mmwr.mm7109a5

**Published:** 2022-03-04

**Figure:**
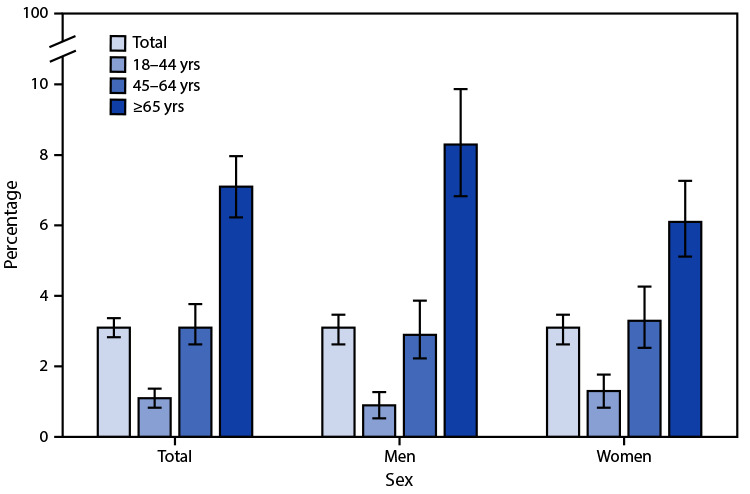
During July–December 2020, 3.1% of adults aged ≥18 years had kidney disease. The prevalence of kidney disease increased with age, from 1.1% among adults aged 18–44 years to 3.1% among those aged 45–64 years and to 7.1% among those aged ≥65 years. Among adults aged ≥65 years, a higher percentage of men had kidney disease (8.3%) compared with women (6.1%). No significant differences were observed by sex for adults aged 18–44 years (0.9% for men versus 1.3% for women) and those aged 45–64 years (2.9% for men versus 3.3% for women).

**Source**: National Center for Health Statistics, National Health Interview Survey, 2020. https://www.cdc.gov/nchs/nhis.htm

For more information on this topic, CDC recommends the following link: https://www.cdc.gov/kidneydisease

